# The Calprotectin Fragment, CPa9-HNE, Is a Plasma Biomarker of Mild Chronic Obstructive Pulmonary Disease

**DOI:** 10.3390/cells14151155

**Published:** 2025-07-26

**Authors:** Mugdha M. Joglekar, Jannie M. B. Sand, Theo Borghuis, Diana J. Leeming, Morten Karsdal, Frank Klont, Russell P. Bowler, Barbro N. Melgert, Janette K. Burgess, Simon D. Pouwels

**Affiliations:** 1 Department of Pathology and Medical Biology, University Medical Center Groningen, University of Groningen, 9713 GZ Groningen, The Netherlandsj.k.burgess@umcg.nl (J.K.B.); 2 Groningen Research Institute for Asthma and COPD, University Medical Center Groningen, University of Groningen, 9713 GZ Groningen, The Netherlands; 3 Nordic Bioscience A/S, 2730 Herlev, Denmark; 4 Department of Pharmacotherapy, Epidemiology, and Economics, Groningen Research Institute of Pharmacy, University of Groningen, 9713 AV Groningen, The Netherlands; frank.klont@rug.nl; 5 Department of Clinical Pharmacy and Pharmacology, University Medical Center Groningen, University of Groningen, 9713 GZ Groningen, The Netherlands; 6 Division of Pulmonary Medicine, Department of Medicine, National Jewish Health, 1400 Jackson Street, Denver, CO 80206, USA; 7 Department of Molecular Pharmacology, Groningen Research Institute of Pharmacy, University of Groningen, 9713 AV Groningen, The Netherlands; 8 Department of Pulmonary Diseases, University Medical Center Groningen, University of Groningen, 9713 GZ Groningen, The Netherlands

**Keywords:** COPD, biomarkers, ECM fragments, CPa9-HNE, S100A9, DAMPs

## Abstract

Chronic obstructive pulmonary disease (COPD) is a chronic inflammatory disease predominantly of the small airways and parenchyma. COPD lungs exhibit an influx of circulating innate immune cells, which, when isolated, display impaired functions, including imbalanced protease secretion. In addition to immune cells, the extracellular matrix (ECM) plays a crucial role in COPD pathology. Remodeling of the ECM can generate ECM fragments, which can be released into circulation and subsequently induce pro-inflammatory responses. COPD is a heterogeneous disease, and serological biomarkers can be used to sub-categorize COPD patients for targeted treatments and optimal recruitment in clinical trials. This study evaluated fragments of calprotectin, collagen type VI, and versican, generated by neutrophil elastase and matrix metalloproteinases (MMP-) 2 and 12, respectively, as potential biomarkers of COPD disease, severity, and endotypes. Lower plasma levels of a neoepitope marker of calprotectin, indicative of activated neutrophils (nordicCPa9-HNETM), were detected in COPD donors compared to controls. CPa9-HNE was associated with milder disease, higher degree of air-trapping, and higher serum levels of MMP-2. Deposition of CPa9-HNE levels in lung tissue revealed no differences between groups. Taken together, CPa9-HNE was found to be a potential marker of mild COPD, but further studies are warranted to validate our findings.

## 1. Introduction

Chronic obstructive pulmonary disease (COPD) is a common lung disease characterized by airflow obstruction and enlarged airspaces [1]. It is associated with chronic inflammation that affects the small airways and parenchymal lung regions. Circulating immune cells, such as neutrophils and monocytes, isolated from patients with COPD, often display a prolonged life span and have impaired functions such as defective chemotaxis, imbalanced secretion of proteases and their inhibitors, and inefficient microbial clearance [2]. A protease called neutrophil elastase, released from neutrophils, is upregulated in COPD and involved in various pathological processes, such as the breakdown of elastin fibers and dysregulation of epithelial cell functions [3,4]. Neutrophil elastase also cleaves calprotectin, a major cytosolic component of neutrophils. Calprotectin belongs to the calcium and zinc-binding S100 protein family and is a heterodimer of S100A8 and S100A9, which can act as damage-associated molecular patterns (DAMPs) upon release into the extracellular space [5].

Pathological regulation of the lung extracellular matrix (ECM) is speculated to play a vital role in COPD [6]. The ECM is a scaffolding network of proteins, proteoglycans, and glycoproteins that support tissue integrity and function and provide essential cues to cells for survival. Collagen and elastin are the most abundant components of the lung ECM but depend on several other proteoglycans and glycoproteins for proper organization and function [7]. The ECM is dynamic and continuously remodeled during homeostasis; however, these processes are often dysregulated in disease. Matrix remodeling can generate ECM fragments [8,9,10,11,12], and while some fragments, such as Proline–Glycine–Proline, released from collagens, are a known chemoattractant, the exact biological function of most remains underexplored [13]. Growing evidence supports their involvement in pro-inflammatory responses, acting as endogenous danger signals or DAMPs, including those generated from aggrecan, biglycan, decorin, tenascin-C, fibrinogen, fibronectin, and versican [14,15,16].

COPD is a heterogeneous disease, and sub-categorization of patients for enhanced specificity and efficiency of care and treatment is essential. Several attempts at identifying biomarkers by investigating blood-cell compositions, serum or sputum protein biomarkers, and metabolic profiles to differentiate COPD endotypes have been performed [17,18,19,20]. However, most biomarkers lack specificity or differentiating capacity. Lately, ECM fragments as markers for (early) diagnostic and prognostic tests have gained popularity. Among patients with COPD, increased plasma levels of calprotectin, associated with decreasing FEV_1_ and a higher risk of mortality, have been reported [21]. Moreover, during COPD exacerbations, circulating levels of S100A8 and S100A9 are also increased [22,23]. With respect to the ECM, specific degradation products of elastin [24], vimentin [25], collagen type I and IV, and the formation product of collagen type V were found associated with COPD [12].

In this study, breakdown fragments of calprotectin generated by human neutrophil elastase (CPa9-HNE) released by activated neutrophils, collagen type VI produced by matrix metalloproteinases (MMP)-2 activity (C6M), and versican digested by MMP-12 (VCANM) were investigated in plasma obtained from control and COPD subjects. The associations between plasma levels of ECM fragments with disease stages and phenotypes, lung function, and serological levels of proteases were investigated. Additionally, ECM protein expression in lung tissue was assessed using immunohistochemistry to understand its relevance in COPD pathology.

## 2. Materials and Methods

### 2.1. Ethics and Study Participants

#### 2.1.1. COPDGene Cohort

Written informed consent was obtained from all participants of the genetic epidemiology of COPD (COPDGene) cohort, and the study was approved by the Institutional Review Board of the National Jewish Health. The study was conducted in accordance with the principles of the Declaration of Helsinki. COPD and non-COPD control donors were selected from the COPDGene cohort as previously described [26]. COPD stages were defined based on the Global Initiative for Chronic Obstructive Lung Disease (GOLD). Briefly, non-COPD control donors (*n* = 125) included never, current, and ex-smokers with an FEV_1_ > 80% predicted and post-bronchodilator FEV_1_/FVC ratio ≥ 0.7, while the COPD donors (GOLD I-IV) were defined by a post-bronchodilator FEV_1_/FVC ratio < 0.7. In the current study, GOLD stages I and II were defined as mild/moderate patients (*n* = 170) and GOLD stages III and IV as severe COPD (*n* = 88). The groups were matched for age, smoking status, and BMI (Table S1).

#### 2.1.2. HOLLAND (Histopathology of Lung Aging and COPD) Cohort

Lung tissue for immunohistochemistry was obtained from control subjects and COPD patients at the University Medical Center Groningen (UMCG, The Netherlands). The study was conducted in accordance with the Research Code of the UMCG (https://umcgresearch.org/w/research-code-umcg accessed on 18 June 2025) and in compliance with national ethical and professional guidelines, Code of Conduct for Health Research (https://www.coreon.org/wp-content/uploads/2023/06/Code-of-Conduct-for-Health-Research-2022.pdf accessed on 18 June 2025). The patient material was not subject to the Medical Research Human Subjects Act in the Netherlands, as confirmed by a statement of the Medical Ethical Committee of the UMCG, and therefore exempt from consent according to national laws (Dutch laws: Medical Treatment Agreement Act (WGBO) art 458/GDPR art 9/UAVG art 24). Any recognizable donor or clinical information was deidentified to the investigators before the beginning of experimental protocols. COPD and non-COPD control donors were selected from the HOLLAND cohort for immunohistochemical staining [27]. COPD and non-COPD control donors were similarly defined as the COPDGene cohort, with an FEV_1_/FVC ratio of <0.7 for patients with COPD and >0.7 for control donors. All donors were ex-smokers and matched for age and sex (Table S1).

### 2.2. Fragment Measurements

Fragments of calprotectin, collagen type VI, and versican were measured in fresh frozen plasma obtained from control and COPD donors. NordicCPa9-HNE^TM^ [28], nordicC6M^TM^ [29], and nordicVCANM^TM^ [30] are degradation fragments generated by the enzymatic activity of neutrophil elastase and MMP-2 and 8, and 12, respectively, all reflecting a disease activity. CPa9-HNE (COPD *n* = 254, control *n* = 125), C6M (COPD *n* = 247, control *n* = 117), and VCANM (COPD *n* = 221, control *n* = 111) were measured using specific competitive enzyme-linked immunosorbent assays (ELISAs). These ELISAs use neo-epitope-specific monoclonal antibodies developed at Nordic Bioscience A/S (Herlev, Denmark). The assays were performed as described previously [28,29,30].

### 2.3. Spirometry and High-Resolution Computed Tomography Scans

Spirometry tests and high-resolution computed tomography (HRCT) scans were conducted on the same day as the plasma collection in subjects from the COPDGene cohort. Spirometry data were collected using the EasyOne spirometer, and CT scans were obtained using multi-detector scanners; detailed protocols are available as published previously [31]. Shortly, volumetric CT acquisitions were obtained on full inspiration (200 mAs), and at the end of normal expiration (50 mAs), images were reconstructed using sub-millimeter slice thickness with the help of smooth and edge-enhancing algorithms. Segmented lung images were used to perform CT phenotyping using Thirona Lung Quantification software (Thirona, Nijmegen, The Netherlands, www.thirona.eu accessed on 18 June 2025). Airway wall thickness (Pi 10) was calculated as the square root of the wall area of a hypothetical airway with an inner perimeter of 10 mm [32]. Low attenuation areas (LAA) less than 950 Hounsfield units (HU) on inspiration and less than 856 HU on expiration were examined for the whole lung. Emphysema was defined if the % LAA less than 950 HU was greater than 6% [31].

Parametric response mapping (PRM) quantified longitudinal changes in the lung by analyzing the density per voxel (3D pixel) within the segmented lung regions [33]. PRM classified CT images as emphysematous when all voxels in the inspiratory CT and expiratory CT were below 950 HU and 856 HU, respectively, as air-trapping when all voxels were above 950 HU in inspiratory CT but below 856 HU in expiratory CT, or as normal when all voxels were above 950 HU and 856 HU in inspiratory and expiratory CT scans, respectively. Furthermore, paraseptal and centrilobular emphysema were visually scored into different severity classifications, including absent, mild, and substantial or absent, trace, mild, moderate, confluent, and advanced destructive, respectively [31].

### 2.4. SOMAscan Assay

Serological levels of parent proteins (from which the ECM fragments originate), including calprotectin, collagen type VI, and versican, were evaluated using aptameric assays of the SOMAscan 1.3 K panel as described previously, and the dataset was reanalyzed in this study [34].

### 2.5. Immunohistochemistry

Formalin-fixed paraffin-embedded (FFPE) control and COPD lung tissues were cut into 6 µM sections. Lung sections were deparaffinized and rehydrated, followed by antigen retrieval for S100A9 and CPa9-HNE using glycine (0.1 M, pH 3.5, 15 min at 100 °C). The sections were cooled to room temperature (RT) and washed thrice with phosphate-buffered saline (PBS). Endogenous peroxidases were blocked by incubating the sections in 0.3% H_2_O_2_ for 30 min. The sections were washed with PBS thrice and incubated with the primary antibodies utilized for nordicCPa9-HNE^TM^ (1:100, Nordic Bioscience, Herlev, Denmark) and S100A9 (1:800, recombinant Anti-S100A9 antibody [EPR3555] (ab92507)) in PBS + 1% bovine serum albumin (BSA) overnight at 4 °C. After incubation, the sections were washed thrice with PBS. Rabbit anti-Mouse Peroxidase (1:100, P0260, Dako, Glostrup, Denmark) or Goat anti-Rabbit Peroxidase (1:100, P0448, Dako, Denmark) in PBS + 1% BSA + 2% human serum albumin were used as secondary antibodies for CPa9-HNE and S100A9, respectively (45 min, RT). After the incubation period, the sections were washed and incubated with Goat anti-Rabbit Peroxidase (1:100) or Rabbit anti-Goat Peroxidase (1:100) as tertiary antibodies (45 min, RT) for CPa9-HNE and S100A9, respectively. The sections were washed, and Vector^®^ NovaRED^®^ substrate (SK-4800, Vector Laboratories, Burlington, Canada) was added for color development. Finally, tissue sections were washed with dH_2_O thrice and counterstained using hematoxylin for 2 min using a standard procedure. The tissue sections were dehydrated, mounted, and later scanned using Hamamatsu Nanozoomer 2.0HT (Hamamatsu Photonic K.K., Shizuoka, Japan) at 40× and viewed using Aperio ImageScope (Leica Biosystems, Nussloch Germany).

### 2.6. Statistics

The differences between age, sex, smoking status, pack-years, BMI, and FEV_1_ in control and COPD groups were tested using Mann–Whitney U or Chi-square tests. The differences in the plasma levels of CPa9-HNE, C6M, and VCANM between control and COPD groups were evaluated using linear regression models corrected for sex and pack-years of smoking. Associations of the plasma levels of fragments with different patient parameters obtained from spirometry and CT scans and serological levels of inflammatory proteins were also examined using linear regression models corrected for sex and pack-years. Univariable linear models were used for subgroup analyses of patients with COPD. Tissue sections stained for S100A9 and CPa9-HNE were analyzed qualitatively. A *p*-value of <0.05 was considered statistically significant.

## 3. Results

### 3.1. Lower Levels of CPa9-HNE Detected in the Plasma of COPD Patients

**Figure 1:**
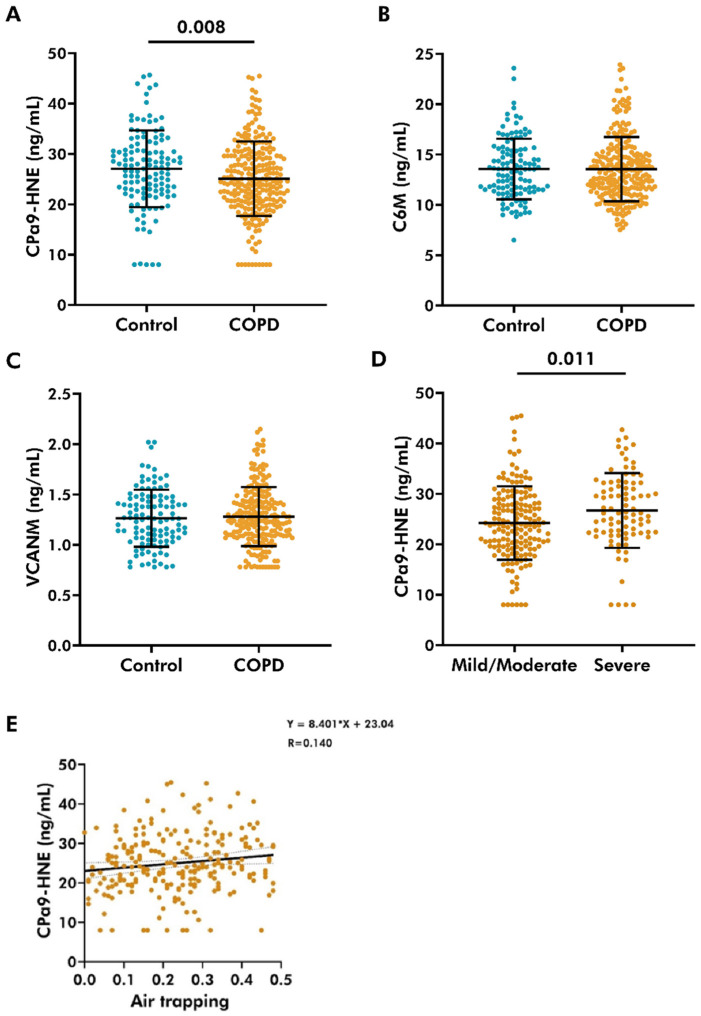
Plasma levels of CPa9-HNE, C6M, and VCANM in controls and patients with COPD. Plasma levels of (**A**) CPa9-HNE (COPD *n* = 254, control *n* = 125), (**B**) C6M (COPD *n* = 247, control *n* = 117), and (**C**) VCANM (COPD *n* = 221, control *n* = 111) were measured in the COPDGene cohort using ELISA. Linear regression models, corrected for sex and pack-years, evaluated the differences between the two groups. (**D**) The association between plasma levels of CPa9-HNE and COPD severity stages (mild/moderate *n* = 170, severe *n* = 88) was evaluated using linear regression analyses. (**E**) Circulating levels of CPa9-HNE were measured using a specialized ELISA in plasma from patients with COPD (*n* = 254). The association between plasma levels of CPa9-HNE and air-trapping was assessed using a linear regression model. A *p*-value lower than 0.05 was considered statistically significant. Graphs have been plotted using mean ± standard deviation.

CPa9-HNE, C6M, and VCANM fragments were measured in plasma from controls and COPD donors. The clinical parameters of donors are available in Tables S1 and S2. Plasma levels of CPa9-HNE were lower in patients with COPD (25.09 ± 7.41 ng/mL (mean ± standard deviation)) compared to controls (27.06 ± 7.61 ng/mL) (*p* = 0.008), as shown in Figure 1. There were no differences in C6M levels between COPD patients (13.57 ± 3.19 ng/mL) and controls (13.56 ± 3.01 ng/mL) (Figure 1B). No differences were found in the plasma levels of VCANM between the COPD (1.28 ± 0.29 ng/mL) and control (1.26 ± 0.28 ng/mL) groups (Figure 1C). Therefore, further analyses were only performed for CPa9-HNE. Analysis of COPD donors based on GOLD severity stages revealed that higher levels of CPa9-HNE were associated with severe COPD (26.71 ± 7.41 ng/mL) compared to mild/moderate disease (24.24 ± 7.28) (*p* = 0.011) (Figure 1D).

### 3.2. Plasma Levels of CPa9-HNE Did Not Associate with Lung Function

As lower CPa9-HNE levels were noted in COPD, the association of plasma levels of CPa9-HNE with lung function was investigated using FEV_1_ and FVC (Table 1). First, the association was tested in all donors, including both healthy controls as well as COPD patients. Secondly, the association was tested using only COPD patients. No associations between CPa9-HNE and FEV_1_ or FVC were found when assessing all donors or when assessing only COPD patients.

### 3.3. Plasma Levels of CPa9-HNE Did Not Associate with Markers of Chronic Bronchitis

After examining the association of circulating levels of CPa9-HNE with lung function, its relevance as a biomarker for subtypes of COPD was explored. First, the association of plasma CPa9-HNE levels with markers of chronic bronchitis was evaluated (Table 1). These parameters included the smoking history of patients and airway wall thickness (Pi10). CPa9-HNE. No associations of CPa9-HNE with these markers were found, neither in all donors nor in COPD patients specifically.

### 3.4. Plasma Level of CPa9-HNE Was Positively Associated with Air-Trapping in COPD

Next, the association of CPa9-HNE with markers of emphysema was studied. These associations were evaluated by assessing the relative lung area with an attenuation of less than 950 HU (LAA < 950), 910 HU, or 856 HU as a measurement of emphysema, mild emphysema, or air-trapping, respectively. However, the plasma levels of CPa9-HNE were not associated with any attenuation measurement (Table 1). However, upon micro-mapping the HRCT scan for voxels associated with air-trapping, emphysema, or normal lung tissue, a weak but statistically significant positive correlation was noted between air-trapping and circulating levels of CPa9-HNE in patients with COPD (Figure 1E). Other measurements evaluating emphysema subtypes, including the severity levels of centrilobular or paraseptal emphysema, did not associate with CPa9-HNE levels (Table 1).

### 3.5. Serological Levels of C-Reactive Protein, S100A9, and Neutrophil Elastase Increased, While MMP-2 Decreased in COPD Patients

**Figure 2:**
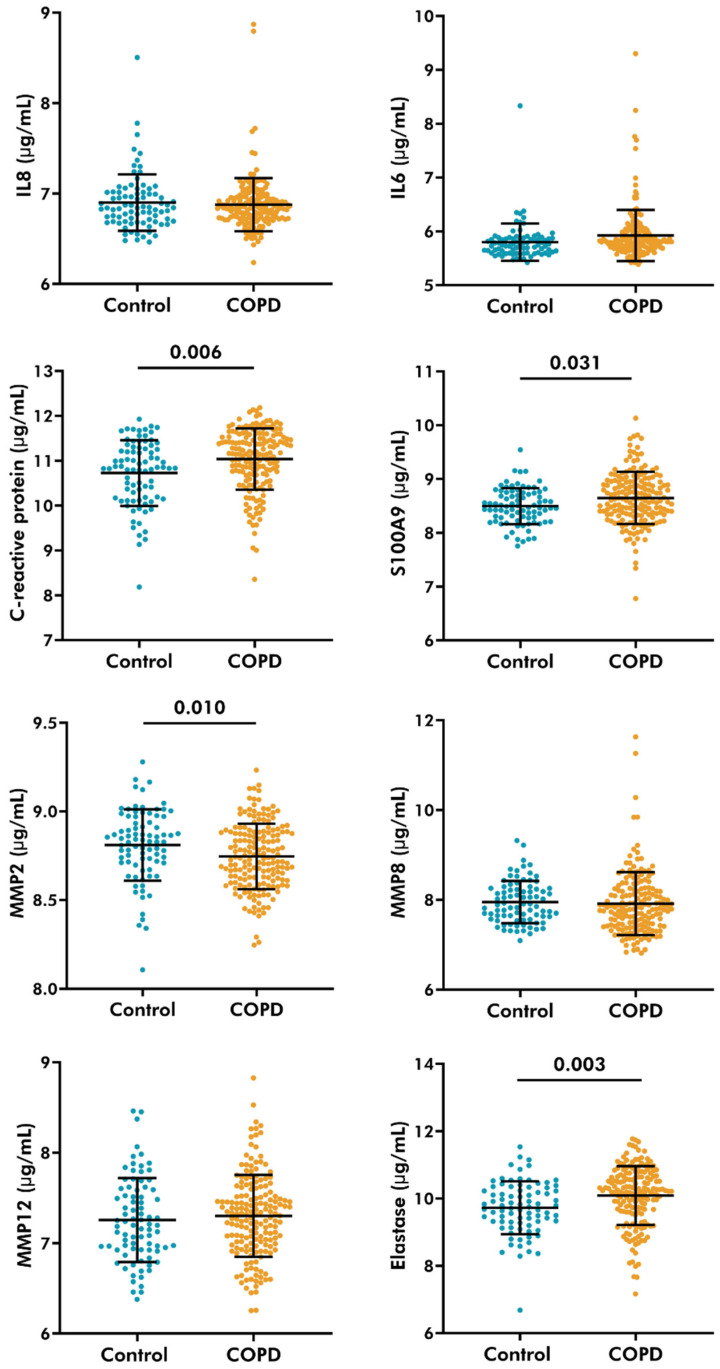
Serological levels of inflammatory cytokines and proteases in control and COPD subjects. IL8, IL6, S100A9, C-reactive protein, MMP 2,8,12, and neutrophil elastase were measured using the SOMAscan assay in COPD (*n* = 258) and control (*n* = 125) subjects of the COPDGene cohort. Linear regression models, corrected for sex and pack-years, were used to evaluate the differences between the two groups. A *p*-value lower than 0.05 was considered significant.

The levels of inflammatory cytokines and proteases were measured in serum obtained from control and COPD donors (Figure 2). Patients with COPD had higher levels of C-reactive protein (11.04 ± 0.68 µg/mL, mean ± standard deviation), S100A9 (8.65 ± 0.49 µg/mL), and neutrophil elastase (10.09 ± 0.87 µg/mL) compared to controls (10.73 ± 0.73 µg/mL, 8.50 ± 0.34 µg/mL, 9.73 ± 0.78 µg/mL, respectively). Furthermore, MMP-2 levels were lower in the serum of COPD patients (8.74 ± 0.19 µg/mL) compared to control subjects (8.83 ± 0.20 µg/mL). No differences were noted in interleukin (IL) 8, 6, MMP-8, or MMP-12 levels between the groups.

### 3.6. Plasma Levels of CPa9-HNE Were Associated with Serological Levels of MMP-2

Next, the relationship between circulating CPa9-HNE levels and serological levels of inflammatory cytokines and proteases was studied. Here, the associations between plasma levels of CPa9-HNE and serological levels of neutrophil elastase, S100A9, MMP-2, MMP-8, MMP-12, and IL8 were assessed. Plasma levels of CPa9-HNE were only associated weakly (*p* = 0.044) with MMP-2 but not with any of the other markers assessed (Table 2).

### 3.7. S100A9 Was Localized to the Parenchyma and the Airway Wall in Lung Tissue

**Figure 3:**
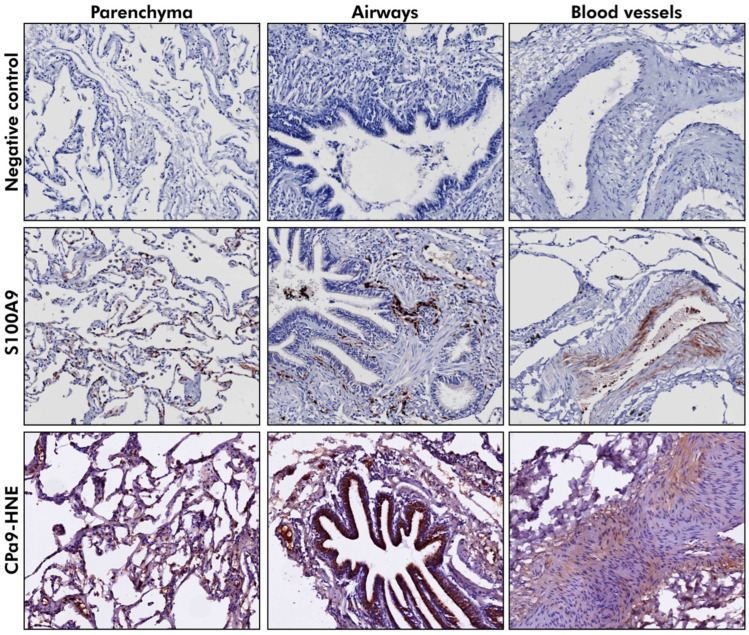
S100A9 and CPa9-HNE were detected in parenchyma, blood vessels, and airways. Control (*n* = 17), moderate COPD (*n* = 13), and severe early-onset (SEO)-COPD (*n* = 12) lung tissues derived from the HOLLAND cohort were stained for S100A9 and CPa9-HNE to study their localization in the parenchyma, airways, and blood vessels within the lung tissue. One SEO-COPD donor tissue was unavailable to stain for S100A9. Hematoxylin stains blue, while Nova red was used to stain S100A9 and CPa9-HNE. Images of positively stained control or COPD lung tissue were captured at 200 µm.

Having detected differences in the plasma levels of CPa9-HNE between controls and patients with COPD, it was of interest to investigate whether CPa9-HNE could be detected in lung tissue (Figure 3). Control (*n* = 17) and COPD (moderate and severe, *n* = 25) lung tissue obtained from the HOLLAND cohort was stained for S100A9 and CPa9-HNE. S100A9 was localized in the parenchyma and parts of the airway wall. On qualitative assessment, a change in the localization of S100A9 from parenchyma to airway walls was noted with increasing severity of COPD (Table 3). In contrast, most tissues had negligible to weak positive staining for CPa9-HNE in the parenchyma, airway, or blood vessel walls. Upon comparing CPa9-HNE staining in control and COPD donors, no differences were noted (Table 3).

## 4. Discussion

Many studies have investigated circulating inflammatory markers in COPD. However, limited data are available on the circulating levels of the enzymatic breakdown products of ECM and inflammatory markers. These enzymatic breakdown products can reflect disease processes, such as a disturbance in the protease–anti-protease balance and tissue remodeling. The current study investigated the differences in circulating levels of protein fragments between control and COPD donors. Fragments generated by neutrophilic activity (CPa9-HNE) and fragments of collagen type VI (C6M) and versican (VCANM) that are of known biological relevance in the pathology of COPD [7] were evaluated as markers of disease. Plasma levels of CPa9-HNE were lower in COPD patients, while for C6M and VCANM, no differences were noted between control and COPD subjects. In patients with COPD, CPa9-HNE was positively associated with air-trapping. Although CPa9-HNE levels were not associated with serological levels of S100A9 or neutrophil elastase, MMP-2 positively correlated with this fragment. This study is also the first to show that CPa9-HNE levels are especially lowered in mild COPD patients, indicating increased enzymatic breakdown during the milder phase of COPD.

Concerning its association with clinical parameters, CPa9-HNE was positively associated with the number of voxels on the HRCT scan mapped for air-trapping, which could indicate the presence of small airway disease. Notably, studies have previously hypothesized that small airway disease could precede the development of chronic bronchitis and emphysema in patients with COPD [35]. Thus, lower CPa9-HNE plasma levels, along with their associations with air-trapping, may arguably suggest that CPa9-HNE could be a marker of milder disease. In contrast with our results, Hansen et al. (2023) reported higher levels of CPa9-HNE in patients with COPD and idiopathic pulmonary fibrosis compared to controls [36]. Although using the same analytical method as our study, there are some key differences between the studies. The study of Hansen et al. assessed a significantly lower number of subjects (39 controls and 67 COPD patients) compared to our study (125 controls and 258 COPD patients), and they also used serum samples while our study used plasma samples. We noted that the lower CPa9-HNE levels in COPD were mainly driven by mild-moderate subjects, suggesting a difference in inflammatory responses in the different stages of COPD. In our study, the COPD patients and controls were matched for age, sex, pack-years, and BMI; however, it is possible that other confounding factors such as medication or the presence of co-morbidities have affected the outcomes.

In this study, higher levels of S100A9, C-reactive protein, and neutrophil elastase were observed in serum from patients with COPD compared to controls, which is consistent with prior research [3,22,37]. Contrary to current knowledge [38], we noted MMP-2 levels were lower in serum obtained from COPD subjects compared to controls. Variations in detection techniques, study population, and subtypes of COPD patients can account, at least partially, for the differences in these results. Furthermore, CPa9-HNE levels positively correlated with MMP-2. While the interaction between calprotectin or S100A9 with MMP-2 in COPD remains unexplored, the regulation of MMP-2 activity by calprotectin has been suggested [39]. Future studies are warranted to identify the biological mechanisms behind the decrease of circulating CPa9-HNE in mild COPD patients and to evaluate why CPa9-HNE associates with specific components of the neutrophilic secretome.

Using an independent cohort, the protein levels of S100A9 and CPa9-HNE were also studied in control and COPD lung tissue. A shift in localization of S100A9 from the parenchyma to airway walls in severe COPD may result from increased extracellular secretion of S100A9 and parenchymal tissue destruction during COPD progression. Compared to S100A9, more donors stained positively for CPa9-HNE in blood vessels. This could indicate secretion into the circulation for clearance and/or perpetuation of immune responses. Positive staining of S100A9 and CPa9-HNE in lung tissue supports that the circulating levels of CPa9-HNE, in part, originate from the lung tissue. However, qualitative analysis of deposited S100A9 and CPa9-HNE was inconclusive, as no differences were noted between control and COPD lungs. This could be a technical limitation of immunohistochemistry and not necessarily reflective of disease processes. However, it may also indicate that the enzymatic breakdown products of calprotectin do not remain in lung tissue but rather spill over into the bloodstream. Moreover, because two different cohorts were used, it was not possible to do a direct comparison between plasma and tissue levels of CPa9-HNE. A limitation of this study is the lack of an independent validation cohort to confirm our results.

Despite a lack of differences in C6M and VCANM fragments between control and COPD groups in the current study, results from other studies have underscored the importance of C6M and VCANM as markers of COPD. Exacerbating COPD patients displayed higher circulating levels of C6M and lower circulating levels of VCANM compared to a follow-up at clinical stability [8]. Moreover, different cohorts have reported higher serological C6M levels associated with decreasing lung function, dyspnea, and risk of mortality [11,40].

Altogether, we observed differences in the circulating levels of protein breakdown products between controls and COPD donors. The discriminative power of CPa9-HNE between controls and patients was limited, decreasing the robustness when using it as a general COPD biomarker. However, we found that circulating CPa9-HNE levels were associated specifically with mild disease and the amount of air-trapping in COPD. Therefore, our observations suggest that CPa9-HNE may play a role in specific aspects of COPD pathophysiology and can potentially be used as a plasma marker of mild disease. Nevertheless, future studies to examine the clinical relevance of our findings are warranted.

## Figures and Tables

**Table 1:** Associations of plasma levels of CPa9-HNE with lung function, chronic bronchitis, and emphysema in the COPDGene cohort.

	Beta	*p*-Value	Lower Bound	Upper Bound	R
FEV1 (% predicted)					
All donors	0.014	0.373	−0.016	0.044	0.092
COPD donors	−0.029	0.175	−0.070	0.013	0.085
FVC (L)					
All donors	−0.080	0.872	−1.051	0.892	0.080
COPD donors	−0.709	0.110	−1.578	0.161	0.101
Smoking					
All donors	0.074	0.934	−1.703	1.852	0.080
COPD donors	1.138	0.291	−0.981	3.256	0.066
Pi10					
All donors	0.134	0.858	−1.330	1.597	0.075
COPD donors	1.483	0.112	−0.350	3.317	0.101
LAA < 950 HU					
All donors	−0.008	0.840	−0.086	0.070	0.075
COPD donors	0.033	0.440	−0.051	0.116	0.049
LAA < 910 HU					
All donors	−0.010	0.663	−0.056	0.036	0.078
COPD donors	0.015	0.590	−0.039	0.069	0.034
LAA < 856 HU					
All donors	0.004	0.855	−0.039	0.047	0.076
COPD donors	0.041	0.099	−0.008	0.091	0.109
Micro-mapping (Normal tissue)					
All donors	−0.511	0.798	−4.433	3.411	0.072
COPD donors	−4.028	0.081	−8.555	0.449	0.115
Micro-mapping (Air-trapping)					
All donors	2.084	0.534	−4.506	8.673	0.079
COPD donors	8.525	0.031	0.778	16.272	0.142
Micro-mapping (Emphysema)					
All donors	−0.682	0.870	−8.868	7.503	0.072
COPD donors	4.248	0.339	−4.481	12.977	0.063
Severity centrilobular score					
All donors	−0.180	0.507	−0.712	0.353	0.082
COPD donors	0.265	0.400	−0.354	0.883	0.054
Severity of paraseptal emphysema					
All donors	0.186	0.722	−0.841	1.213	0.076
COPD donors	0.216	0.705	−0.910	1.342	0.024

Linear regression analysis was used to evaluate the associations between plasma levels of CPa9-HNE and FEV_1_, FVC, smoking status, Pi10 (airway wall thickness), and markers of emphysema in all subjects (*n* = 379) and patients with COPD (*n* = 254) of the COPDGene cohort. A *p*-value lower than 0.05 was considered significant. FEV_1_ = forced expiratory volume in 1 s; FVC = forced vital capacity. LAA = lower attenuation areas; HU = Hounsfield units. HRCT scans were micro-mapped to assess voxels associated with normal lung tissue, air-trapping, or emphysematous tissue.

**Table 2:** Associations of plasma levels of CPa9-HNE with serological levels of other proteins released by immune cells during inflammation.

	Beta	*p*-Value	Lower Bound	Upper Bound	R
Elastase					
All donors	0.085	0.878	−1.005	1.175	0.100
COPD donors	−0.052	0.937	−1.368	1.263	0.006
S100A9					
All donors	−0.646	0.546	−2.752	1.459	0.106
COPD donors	−0.547	0.650	−2.921	1.827	0.035
IL8					
All donors	0.011	0.994	−3.138	3.160	0.099
COPD donors	−0.329	0.869	−4.250	3.250	0.013
MMP2					
All donors	4.989	0.044	0.135	0.062	0.160
COPD donors	6.339	0.044	0.164	12.515	0.153
MMP8					
All donors	0.149	0.844	−1.341	1.639	0.100
COPD donors	0.591	0.480	−1.057	2.240	0.054
MMP12					
All donors	−1.119	0.307	−3.274	1.036	0.118
COPD donors	−0.683	0.609	−3.311	1.945	0.039

Linear regression analysis was used to evaluate the associations between plasma levels of CPa9-HNE and serological levels of human neutrophil elastase, S100A9, and IL8 were examined using linear regression analysis in all donors (*n* = 379) and COPD donors (*n* = 254) of the COPDGene cohort. A *p*-value lower than 0.05 was considered significant.

**Table 3:** Qualitative assessment of localization of S100A9 and CPa9-HNE in control and COPD lung tissue derived from the HOLLAND cohort.

Compartment	Staining Intensity	Control (% Donors)	COPD Moderate (% Donors)	COPD Severe (% Donors)
S100A9				
Parenchyma	No-weak	47.06	69.23	72.73
	Moderate	29.41	0	9.10
	Strong	23.53	30.77	18.18
Airway wall	No-weak	47.06	38.46	36.36
	Moderate	23.52	23.08	9.10
	Strong	29.41	38.46	54.55
Blood vessels	No-weak	100	92.3	100
	Moderate	0	0	0
	Strong	0	7.70	0
CPa9-HNE				
Parenchyma	No-weak	70.59	92.31	83.33
	Moderate	5.88	7.69	8.33
	Strong	23.53	0.00	8.33
Airway wall	No-weak	52.94	84.62	83.33
	Moderate	29.41	7.69	16.67
	Strong	11.76	7.69	0.00
Blood vessels	No-weak	76.47	69.23	91.67
	Moderate	17.65	30.77	8.33
	Strong	5.88	0.00	0.00

## Data Availability

All data are presented in this manuscript or in the Supplementary data document. Raw data can be obtained upon reasonable request from the corresponding author.

## Supplementary Materials

The following supporting information can be downloaded at: https://www.mdpi.com/article/10.3390/cells14151155/s1, Table S1: Clinical characteristics of donors included from the COPDGene and HOLLAND cohort; Table S2: Characteristics of COPD donors included in this study.
