# Supplementary material for: The Calprotectin Fragment, CPa9-HNE, Is a Plasma Biomarker of Mild Chronic Obstructive Pulmonary Disease
Source: Cells. 2025 Jul 26;14(15):1155. doi: 10.3390/cells14151155 (PMC12346652; doi:10.3390/cells14151155)
Supplement: Supplementary file 1 [file cells-14-01155-s001.zip › cells-3738381-supplementary.pdf]

TITLE: The calprotectin fragment, CPa9-HNE, is a plasma biomarker of mild chronic obstructive pulmonary disease

Authors: Mugdha M Joglekar[1,2], Jannie MB Sand[3], Theo Borghuis[1], Diana J Leeming[3], Morten Karsdal[3], Frank Klont[4,5], Russell P Bowler[6], Barbro N Melgert[2,7], Janette K Burgess[1,2], Simon D Pouwels[1,2,8]

Affiliations:
[1]University of Groningen, University Medical Center Groningen, Department of Pathology and Medical Biology, Groningen, The Netherlands

[2]University of Groningen, University Medical Center Groningen, Groningen Research Institute for Asthma and COPD, Groningen, The Netherlands

[3]Nordic Bioscience A/S, Herlev, Denmark

[4]University of Groningen, Department of PharmacoTherapy, Epidemiology, and Economics, Groningen Research Institute of Pharmacy, Groningen, The Netherlands

[5]University of Groningen, University Medical Center Groningen, Department of Clinical Pharmacy and Pharmacology, Groningen, The Netherlands

[6]Division of Pulmonary Medicine, Department of Medicine, National Jewish Health, 1400 Jackson Street, Denver, CO, 80206, USA

[7]University of Groningen, Groningen Research Institute of Pharmacy, Department of Molecular Pharmacology, Groningen, Netherlands

[8]University of Groningen, University Medical Center Groningen, Department of Pulmonary Diseases, Groningen, The Netherlands

Supplementary Material

Table S1: Clinical characteristics of donors included from the COPDGene and HOLLAND cohort.

	COPDGene cohort			HOLLAND cohort		
	**Control (n= 125)**	**COPD (n= 258)**	**p-value**	**Control (n= 17)**	**COPD (n= 24)**	**p-value**
**Age (years, median, range)**	65.70 (55.00 – 79.00)	65.20 (45.00 - 81.00)	0.880	62.00 (43.00 – 76.00)	67.00 (47.00 – 81.00)	0.700
**Sex (% female)**	58.40	46.10	**0.024**	53.00	41.70	0.476
**Smoking (% smokers)**	23.20*	24.80*	0.731	100.00[&]	100.00[&]	-
**Pack years (median, range)**	35.00 (10.00 – 145.00)	46.20 (10.00 – 180.00)	**3.390×10^{-7}**	20.00 (1.50 – 52.50)[#]	30.00 (6.00 – 65.00)[##]	0.230
**BMI (kg/m^2, median, range)**	28.90 (19.00 – 44.00)	27.50 (18.00 – 46.00)	0.060	-	-	-
**FEV_1 (% predicted, median, range)**	97.40 (80.00 – 150.00)	59.40 (14.00 – 135.00)	**1.660×10^{-44}**	102.20 (75.60 – 133.00)[$]	50.70 (12.00 – 75.40)	**7.960×10^{-11}**

*Mann-Whitney U (age, pack years, BMI and FEV_1) or Chi-Square (sex and smoking status) tests were used to evaluate the differences between control and COPD donors. A p-value lower than 0.05 was considered statistically significant. Significant values are highlighted in bold. *The smoking status of smokers is unknown (current versus ex-smokers). [&]All donors are ex-smokers. [#]Pack years are missing for five donors and [##]1 donor respectively. BMI data was not available for the HOLLAND cohort. [$]$FEV_1$ measurements were unavailable for two donors. BMI = body mass index; FEV_1 = forced expiratory volume in one second.*

Table S2: Characteristics of COPD donors included in this study.

	COPDGene cohort- COPD donors		
	**Mild/moderate (n= 167)**	**Severe (n=87)**	**p-value**
**Age (years, median, range)**	65.10 (45.00- 81.00)	65.00 (47.00- 79.00)	0.557
**Sex (% female)**	47.90	44.83	0.641
**Smoking (% smokers)**	26.95	20.70	0.273
**Pack years (years, median, range)**	44.00 (10.00- 118.00)	48.80 (17.00- 180.00)	0.083
**BMI (kg/m2, median, range)**	27.50 (18.00- 44.00)	27.80 (19.00- 46.00)	0.487
**FEV_1 (% predicted, Median, range)**	71.20 (50.00- 135.00)	38.40 (14.00- 50.00)	**4.630×10^{-39}**

*Mann Whitney U (age, pack years, BMI, and FEV_1) or Chi-square (sex and smoking status) tests were used to compare the donor characteristics of the mild/moderate (n= 167) and severe (n= 87) COPD groups. The smoking status of smokers is unknown (current versus ex-smokers). A p-value of <0.05 was considered significant. BMI = body mass index; FEV_1 = forced expiratory volume 1. *Pack year was unavailable for one severe COPD donor.*
